# Susceptibility of *Dalotia coriaria* (Kraatz) (Coleoptera: Staphylinidae) to Entomopathogenic Nematodes (Rhabditida: Heterorhabditidae and Steinernematidae)

**DOI:** 10.3390/insects6010224

**Published:** 2015-03-18

**Authors:** Joseph Tourtois, Matthew J. Grieshop

**Affiliations:** Department of Entomology, Michigan State University, East Lansing, MI 48824, USA; E-Mail: riddlej2@msu.edu

**Keywords:** *Dalotia (= Atheta) coriaria*, entomopathogenic nematodes, intraguild predation

## Abstract

*Dalotia coriaria* (Kraatz) (Coleoptera: Staphylinidae) and entomopathogenic nematodes (Rhabditida: Heterorhabditidae and Steinernematidae) are two soil-dwelling biological control agents used to manage western flower thrips, *Frankliniella occidentalis* (Pergande) (Thysanoptera: Thripidae) and fungus gnats *Bradysis spp.* (Diptera: Sciaridae) in glasshouses. Growers often use multiple natural enemies to achieve economic control, but knowledge of interactions among natural enemies is lacking. We conducted a laboratory bioassay to test the pathogenicity of four commercially available nematode species—*Heterorhabditis bacteriophora* Poinar (Rhabditida: Heterorhbditidae), *Steinernema carpocapsae* (Weiser) (Rhabditida: Steinernematidae), *S. feltiae* (Filipjev), and *S. riobrave* Cabanillas *et al.*—to third instar and adult *D. coriaria*. Third instars were three times more susceptible than the adults to the entomopathogenic nematodes. Mortality for *D. coriaria* adults and third instars treated with *S. feltiae* and *H. bacteriophora* was lower than the mortality for *D. coriaria* adults and third instars treated with *S. carpocapsae* and *S. riobrave*. Neither infective juvenile foraging behavior nor size correlates with *D. coriaria* mortality. *Dalotia coriaria* appears to be most likely compatible with applications of *S. feltiae* and *H. bacteriophora*.

## 1. Introduction

*Dalotia coriaria* (Kraatz) (Coleoptera: Staphylinidae) and entomopathogenic nematodes are two soil-dwelling biological control agents used to manage common greenhouse pests including: thrips, fungus gnats, and shore flies. The use of biological control in greenhouses has become more desirable due to the development of pesticide resistance in pest populations [1]. Growers often use multiple natural enemies to achieve economic control but knowledge of interactions among natural enemies is lacking.

Western flower thrips, *Frankliniella occidentalis* (Pergande) (Thysanoptera: Thripidae) is a major pest of vegetable production and floriculture in greenhouses and nurseries [2,3]. Due to their cryptic behaviors (eggs laid in plant tissue, pupation occurs in the soil, and feeding on developing tissues) and their resistance to many insecticides, biological control has become increasingly important to successful western flower thrips management programs [2,3,4,5]. Both entomopathogenic nematodes and *D. coriaria* are employed to target the soil-dwelling stage of western flower thrips (*i.e.,* prepupae and pupae).

Fungus gnats *Bradysis spp.* (Diptera: Sciaridae) are also major pests in greenhouses. Fungus gnat larvae feed on many plant roots. Nematodes and *D. coriaria* are recommended soil-dwelling biological control agents to target this pest. Five species of Heterorhabditis and four species of Steinernema have been tested for controlling fungus gnats [6,7,8]. *Steinernema feltiae* is often the leading performer in controlling fungus gnats. Both larval and adult *D. coriaria* feed on fungus gnat eggs and larvae [9].

*Dalotia coriaria* is a small (3–4 mm), highly mobile, soil-dwelling polyphagous predator. Larvae are a pale yellow to cream color; and the adults are a glossy, dark color [10]. The body posture of the adults is typically S-shaped with their heads pointed down and their abdomens upturned. Both the larvae and adults are polyphagous—feeding on multiple life stages of mites and other insects [9,10,11].

Adult *D. coriaria* are voracious predators and have been shown to consume as many as 95 second instar thrips, 78 thrips pupae, 154 fungus gnat eggs, or 150 first instar fungus gnats within 24 h [9]. Third instar beetles can consume an equally impressive 100 eggs and 100 first instar fungus gnats in a 24 h period [9]. In a laboratory bioassay, one adult rove beetle can consume 68%–78% of the second and third instar fungus gnats presented to them in Petri dishes within 24 h [12].

In a screened greenhouse trial with *Impatiens* (L.) (Ericales: Balsaminaceae), *D. coriaria* reduced western flower thrips populations by 53%–82% [13]. On caged *Gerbera jamesonii* (Bolus ex Hook) (Asterales: Asteraceae) and *Chrysanthemums spp.* (Asterales: Asteraceae), *D. coriaria* did not reduce western flower thrips populations with thrips ranging from 1–1.8 to 6.9–7 on *G. jamesonii* and *Chrysanthemums* spp., respectively [5]. In a field experiment with five or 10 adult rove beetles per caged parsley *Petroselinum crispum* (Mill.) Nyman ex A. W. Hill (Apiales: Apiaceae) pot, there were 75% and 85% fewer fungus gnat adults on yellow sticky cards compared to the control over a 22 d period [13]. In a follow-up experiment, two adult rove beetles reduced the number of fungus gnats on yellow sticky cards by 48% [14].

Entomopathogenic nematodes in the families Heterorhabditidae and Steinernematidae are soil dwelling round worms that are obligate parasites of insects. Infective juveniles (IJ) (or dauer larvae) enter insect hosts through natural openings—mouth, anus, and spiracles. Inside the insect haemocoel, the juvenile nematodes release their symbiotic bacteria and kill the host within 24–48 h. Nematodes complete their development within the host, proceeding through one to three generations. When host resources are depleted, thousands of infective juveniles emerge from the cadaver in search of a new host [15]. *Heterorhabditis bacteriophora* Poinar (Rhabditida: Heterorhbditidae), *Steinernema carpocapsae* (Weiser) (Rhabditida: Steinernematidae), *S. feltiae* (Filipjev), and *S. riobrave* Cabanillas *et al.* are four commonly available entomopathogenic nematodes.

*Heterorhabditis bacteriophora*, *S. feltiae*, and *S. carpocapsae* can infect > 50% of second instar thrips and prepupae and 11%–54.5% of the pupae [16]. *Heterorhabditis* (12 strains) and 16 strains of *Steinernema* have been screened for infectivity in western flower thrips in several laboratory studies [16,17,18,19]. Thrips mortality ranged from 0 to 75%. Chyzik *et al.* (1996) found that *H. bacteriophora* HP88 was the most effective at reducing western flower thrips populations by 39%. Ebssa *et al.* (2001) reported > 50% thrips prepupae mortality for several nematode strains. They chose *H. bacteriophora* HK3, *S. feltiae* Sylt, and *S. carpocapsae* DD136 as the strain that caused the highest mortality for each species. Premachandra *et al.* (2003) chose *H. bacteriophora* HK3 and *S. feltiae* Nemaplus^®^ as the most effective nematodes. Ebssa *et al.* (2004) identified *H. indica* Poinar, Karunakar and David and *S. bicornutum* Tallosi, Peters and Ehlers as the nematode species from each genus for causing the highest western flower thrips mortality. Thus, multiple nematode species and strains in both genera can be used for thrips management.

Entomopathogenic nematode host searching strategy is generally considered to be on a continuum from cruiser to ambusher [15,20]. Cruisers such as *H. bacteriophora* move through the soil in search of a host [21]. Ambushers like *S. carpocapsae*, perform a behavior known as nictitating, where they elevate 95% of their body and wave back and forth, waiting for a host to pass by [22]. Nematodes such as *S. feltiae* and *S. riobrave* are intermediate in their search strategy and display both behaviors [23]. Ambushers are often applied to target mobile hosts and cruisers are often applied to target stationary hosts. Thus, it is possible that *D. coriaria*, which is highly mobile, may more likely be infected by an ambusher, *S. carpocapsae*, than the cruiser, *H. bacteriophora*.

*Dalotia coriaria* and entomopathogenic nematodes are both soil-dwelling organisms used as biological control agents to manage the same pests, *i.e.* western flower thrips, fungus gnats, and shore flies; therefore, both could be applied at the same time and come into contact with each other. To date a single study has tested the compatibility of *D. coriaria* with one species of nematode, *S. feltiae*, and concluded that the two organisms are likely compatible for simultaneous release [11]. Biological control companies recommend the use of *S. feltiae* to control both thrips and fungus gnats; however, other nematode species have also been shown to be effective against western flower thrips. A handful of laboratory studies screened multiple nematode species against western flower thrips and found that nematode species such as *H. bacteriophora*, *H. indica*, and *S. bicornutum* killed similar numbers or more thrips prepupae than *S. feltiae* [16,17,18,19]. Jandricic *et al.* (2006) showed that *S. feltiae* is capable of infecting third instar *D. coriaria* in a laboratory bioassay, but only 16% of the mortality confirmed nematode infection at the highest dose—50 infective juveniles (IJ)/cm^2^. In a microcosm bioassay, 100 IJ/cm^2^ caused 25% mortality of third instars [11]. The objective of this study was to determine the susceptibility of *D. coriaria* third instars and adults to four commonly used species of entomopathogenic nematodes: *H. bacteriophora*, *S. carpocapsae*, *S. feltiae*, and *S. riobrave*.

## 2. Experimental Section

We conducted a 4 × 3 × 2 factorial experiment to test the pathogenicity of four nematode species—*H. bacteriophora*, *S. carpocapsae*, *S. feltiae*, and *S. riobrave*—at multiple doses—one-half, one, and two times the recommended rate of application [24]—for two life stages of *D. coriaria*—third instar and adult. There were 24 treatments plus two controls—adult and third instar beetles without nematodes.

### 2.1. Insect Rearing and Nematode Cultures

We established laboratory colonies of *D. coriaria* with beetles purchased from BioBest (Leamington, ON, Canada) and Syngenta (Little Clacton, UK). Beetles used for this study were either laboratory reared or purchased from IPM Laboratories Inc. (Locke, New York, NY, USA). Laboratory colonies were reared in two different sized plastic containers. One plastic container was a 2.25 L rectangle box from Ziploc (Racine, WI, USA). We drilled two ventilation holes (dia 2.54 cm) into the lid and covered the holes with bridal veil. Since four of these containers did not supply enough beetles for the experiment, we increased *D. coriaria* rearing production with four additional larger (9.4 L capacity) rectangular plastic containers from Rubbermaid (High Point, NC, USA). We drilled two ventilation holes (dia 7.62 cm) into the lid and covered the holes with bridal veil. Both containers contained grounded coconut husk (coir) (Canna Continental, Los Angeles, CA, USA) and vermiculite (Good Earth Horticulture, Inc. Lancaster, NY, USA) (50:50 ratio) as a substrate. The smaller container held 1 L of substrate, and the larger container held 6 L of substrate. We kept containers on a laboratory bench under ambient conditions (22.0 ± 0.9 °C, 47.9% ± 18.4% RH) near windows and subjected to the natural light cycle. We feed the beetles certified organic chicken feed (HiLo Acres, Portland, MI, USA) on a weekly schedule—15 mL to the smaller and 30 mL to the larger containers. Chicken feed was mixed into the media and water was added as needed to maintain moisture [13,14].

*Heterorhabditis bacteriophora* Oswego strain was obtained from a laboratory culture (Anne Nielsen, Rutgers Agriculture Research and Extension Center, Bridgeton, NJ, USA). *Steinernema carpocapsae* and *S. feltiae* were obtained from BeckerUnderwood (Ames, IA, USA). *Steinernema riobrave* 355 strain was obtained from David Shapiro-Illan, USDA-ARS, Byron, GA, USA. The four nematode species were reared on late instar *Galleria mellonella* (L.) (Lepidoptera: Pyralidae) in laboratory colonies. Five *G. mellonella* were placed on filter paper in an inverted Petri dish and infected with 500 infective juveniles (IJ) in aqueous solution [25]. Infective juveniles were harvested using a White trap [26] and stored in 600 mL tissue culture flasks with vented cap (Corning Inc., Tewksbury, MA, USA) in the dark, under ambient laboratory conditions. We deposited beetle and nematode voucher specimens in the Michigan State University A.J. Cook Arthropod Research Collection (voucher number 2014–08).

### 2.2. Experimental Methods

The test arena consisted of a 1.7 mL microcentrifuge tube (Denville Scientific Inc., South Plainfield, NJ, USA) with a hole (approx. 0.045 mm) in the lid to allow air exchange [27]. We cut a piece of No. 1 Whatman filter paper (dia 55 mm) into eight equal radial slices. We inserted a slice into each tube to provide a substrate for nematodes and to help regulate relative humidity. We added one grain of organic rolled oats to each tube as supplemental food for the beetles. Infective juveniles were applied in aqueous solution (50 μL) to the filter paper and one beetle was added per tube [27].

The recommended rate of nematode application is 100 IJ/cm^2^ for western flower thrips management [24] and the cap of the microcentrifuge tubes used in the experiment were approximately 1 cm^2^. Thus, one-half, one, and two times the recommended rate was calculated at 50 IJ/cm^2^, 100 IJ/cm^2^, 200 IJ/cm^2^. Infective juveniles were used within 14 d of harvest from laboratory colonies.

Availability of sufficient beetles determined experimental timing. From June to August 2014, two to four replicates were set-up and run at a time, until 20 replicates were completed. In each replicate, there was one tube for each treatment and four tubes each for adult and third instar controls. We assessed nematode viability for each run by infecting *G. mellonella*. For each nematode species, we placed eight *G. mellonella* in an inverted 9 cm Petri dish with 160 infective juveniles. Arenas and Petri dishes were placed in a growth chamber set at 24.4 ± 0.3 °C, 92.7% ± 15.2% RH, with 24 h darkness. Beetle mortality was assessed daily for 4 d. The experiment was terminated on the fourth day due to excessive fungal growth in the arenas. All the tubes were placed in the freezer (−20 °C). We dissected the dead beetles to check for the presence of nematodes.

### 2.3. Statistical Analysis

We excluded the last four replicates of beetles exposed to *H. bacteriophora* from the analysis because the *G. mellonella* were poorly infected (*i.e.,* < 88% infection). We excluded three tubes each of adult and larval beetles in the control from one replicate from the analysis since holes were not punctured into the lid of the microcentrifuge tubes. Lastly, we excluded one entire block from analysis since nematodes were found in the dead beetles in the control treatments.

Since each treatment has represented only once in each block (replicate), we could not calculate a corrected mortality for each replicate. Our data was binary and collected for multiple days, thus we utilized survival analysis. Survival analysis was tested with Cox’s proportional hazard function using PROC PHREG in SAS 9.3 [28,29]. Beetle mortality was modeled by insect stage, nematode species, dose rate, and interaction terms. Terms with a *p*-value > 0.15 were dropped from the model. A logistic regression was performed using the R statistical language [30] to compare the number of infected beetles per treatment. Beetle infection was modeled by insect stage, nematode species, dose rate, and interaction terms. The step function was used to select a reduced model based on the lowest AIC value (Table 1). Multiple comparisons of the slopes were conducted using the contrast package [31].

**Table 1:** Model selection based on AIC values using the step function in R 3.1.1.

Model	AIC
EPN + Rate + stage + EPN:Rate + EPN:stage + Rate:stage + EPN:Rate:stage + Block	263.33
EPN + Rate + stage + EPN:Rate + EPN:stage + Rate:stage + EPN:Rate:stage	255.15
EPN + Rate + stage + EPN:Rate + EPN:stage + Rate:stage	252.82
EPN + Rate + stage + EPN:Rate + EPN:stage	250.80
EPN + Rate + stage + EPN:Rate	249.30

EPN = entomopathogenic nematode, Rate = dose of infective juveniles, stage = developmental stage of *D. coriaria*.

## 3. Results

### 3.1. Beetle Mortality

**Figure 1:**
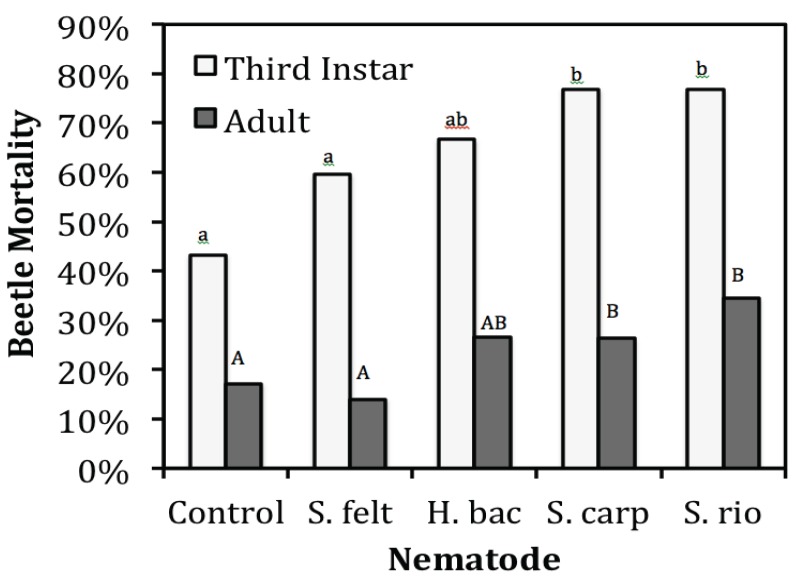
Percent mortality of *D. coriaria* on Day 4. Since dosage was not significant (χ^2^ = 5.16, df = 2, *p* = 0.076), data for dosage was combined for each nematode species. H. bac = *H. bacteriophora*, S. carp = *S. carpocapsae*, S. felt = *S. feltiae*, S. rio = *S. riobrave*. Bars with different letters are significantly different (*p* < 0.05).

Third instar *D. coriaria* are approximately three times more susceptible to the nematodes than the adults (χ^2^ = 77.54, df = 1, *p* < 0.001). In the control, there was 17% adult beetle mortality and 43% mortality for the third instars (Figure 1). The main effect of nematode species was significant (χ^2^ = 13.54, df = 4, *p* = 0.009). The dosage rate of the nematodes was not significant (χ^2^ = 5.16, df = 2, *p* = 0.076). Mortality for *D. coriaria* adults and third instars treated with *S. feltiae* and *H. bacteriophora* was not significantly different from the control (χ^2^ = 0.03, df = 1, *p* = 0.873 and χ^2^ = 2.084, df = 1, *p* = 0.149, respectively) (Figure 1). Mortality for *D. coriaria* adults (26%) and third instars (77%) treated with *S. carpocapsae* was significantly higher than the control (Figure 1) (χ^2^ = 6.24, df = 1, *p* = 0.013) and *S. feltiae* (χ^2^ = 7.06, df = 1, *p* = 0.008), but not *H. bacteriophora* (χ^2^ = 1.22, df = 1, *p* = 0.269). Mortality for *D. coriaria* adults (34%) and third instars (77%) treated with *S. riobrave* was significantly higher than the control (Figure 1) (χ^2^ = 6.38, df = 1, *p* = 0.012) and *S. feltiae* (χ^2^ = 7.09, df = 1, *p* = 0.008), but not significantly different from *H. bacteriophora* (χ^2^ = 1.18, df = 1, *p* = 0.278) or *S. carpocapsae* (χ^2^ = 0.002, df = 1, *p* = 0.965). None of the interaction terms were significant.

### 3.2. Presence of Nematodes in Cadavers

**Figure 2:**
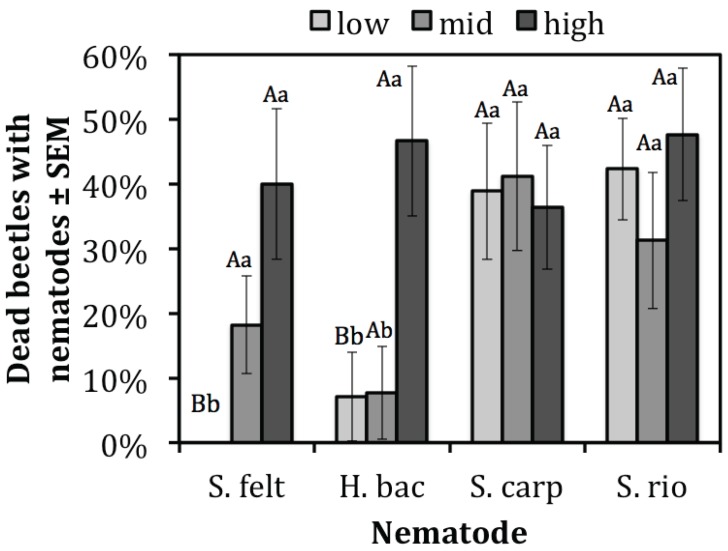
Percent dead *D. coriaria* with confirmed nematodes. H. bac = *H. bacteriophora*, S. carp = *S. carpocapsae*, S. felt = *S. feltiae*, S. rio = *S. riobrave.* Within each nematode group, bars with different lowercase letters are significantly different (*p* < 0.05). For each rate across nematode species, bars with different uppercase letters are significantly different (*p* < 0.05).

Not all of the dead beetles contained nematodes. Even though third instar mortality was higher in the presence of entomopathogenic nematodes than adult mortality, a similar number of nematodes were recovered from both third instars and adults (χ^2^ = 237, df = 197, *p* = 0.121). The main effects of nematode species and dose were significant for the number of nematodes recovered from adults and third instars (χ^2^ = 246, df = 200, *p* = 0.025 and χ^2^ = 240, df = 198, *p* = 0.046, respectively). The two-way interaction term for nematodes species and rate was also significant (χ^2^ = 223, df = 191, *p* = 0.028). There was an increasing dosage effect for *S. feltiae* and *H. bacteriophora* but not *S. carpocapsae* and *S. riobrave*. Nematodes were recovered from 0, 18%, and 40% of the adult and larval beetles that died after being treated with *S. feltiae* at the low, intermediate, and high rates, respectively, with significantly more nematodes recovered at the high rate relative to the low rate (t = 2.13, df = 191, *p* = 0.034) (Figure 2). For *H. bacteriophora*, nematodes were found in 7%, 8%, and 47% of the dead adult and larval beetles treated at the low, intermediate, and high rates, respectively, with significantly more nematodes recovered from the high rate relative to the low and intermediate rates (t = 2.13, df = 191, *p* = 0.034 and t = 1.98, df = 191, *p* = 0.049, respectively) (Figure 2). For the *S. carpocapsae* treatment, nematodes were found in 39%, 41%, and 36% of the dead adult and larval beetles treated at the low, intermediate, and high rates, respectively, without any significant differences between rates (Figure 2). For the *S. riobrave* treatment, nematodes were found in 42%, 31%, and 48% of the dead adult and larval beetles treated at the low, intermediate, and high rates, respectively, without any significant differences between rates (Figure 2).

## 4. Discussion

Entomopathogenic nematodes and *D. coriaria* are soil-dwelling biological control organisms that are likely to encounter each other, especially when used as part of an augmentative biological control program. A previous study tested the laboratory susceptibility of *D. coriaria* to only one nematode, *S. feltiae*, and concluded that third instar mortality is dose dependent but not adult beetle mortality [11]. The four nematode species tested in the present study were able to infect third instar and adult *D. coriaria* with varying success, but only *S. carpocapsae* and *S. riobrave* significantly increased mortality (Figure 1). Adult beetles were less susceptible than third instars, a pattern seen in other beetle hosts. *Dalotia coriaria* adults and third instars were less susceptible to *S. feltiae* than the other three species. Thus, *S. feltiae* appears to be a good candidate to use with *D. coriaria* in biological control programs of greenhouse pests.

In their laboratory studies, Jandricic *et al.* (2006) showed that adult *D. coriaria* was not susceptible to *S. feltiae* but third instar mortality was dose dependent. Mortality at the highest dose rate of 50 IJ/cm^2^ was 27%, which was significantly greater than the two lower rates and control [11]. In contrast the present study did not show a dosage effect for the mortality. This inconsistency may be due to the higher doses tested. In Jandricic *et al.* (2006), third instars were treated with 12 IJ/cm^2^, 25 IJ/cm^2^, and 50 IJ/cm^2^. Whereas, in this study, the doses were 50 IJ/cm^2^, 100 IJ/cm^2^, and 200 IJ/cm^2^ and resulted in higher mortality, 74%, 47%, and 58% for each dose respectively.

Third instars were two to four times more likely to die than the adults. For both phytophagous and predatory beetles, adults are typically less susceptible to nematode infection, but not always [32]. Adult carrot weevil *Listronotus oregonensis* (LeConte) (Coleoptera: Curculionidae) is less susceptible to *H. heliothidis* (Khan, Brooks, and Hirschmann), *S. bibionis* (Steiner), and *S. carpocapsae* DD-136 than third instars [33]. Adult lesser mealworm *Alphitobius diaperinus* (Panzer) (Coleoptera: Tenebrionidae) is less susceptible to *S. carpocapsae* DD-136 than late instars, but not *H. heliothidis* or *S. glaseri* (Steiner) [34]. Larvae of the confused flour beetle *Tribolium confusum* du Val (Coleoptera: Tenebrionidae) are generally more susceptible to *S. feltiae* than the adults [35]. Multiple adult predatory beetles including *Philonthus sp.* (Coleoptera: Staphylinidae) were less susceptible to *H. bacteriophora* and *S. carpocapsae* than last instars [36]. It is not known why adults are generally less susceptible than larvae, but it could be due to cuticle thickness, morphological differences in body openings, or behavior [36]. *Dalotia coriaria* third instar mortality was 43% in our control, this was likely due to higher susceptibility to opportunistic fungi compared to adults. Whether this is a typical mortality rate for this life stage has not been determined.

Insect mortality due to entomopathogenic nematodes is correlated with behavior and morphology of both the host and nematode [22,37]. Infective juveniles that cruise are better adopted to search for sedentary hosts; whereas, ambushers are better adopted to search for mobile hosts at the soil surface. Spiracles smaller than infective juvenile body width [38] or fitted with sieve plates restrict nematodes entry [39]. In the present study, neither search strategy nor size correlates with beetle mortality. Aspects of beetle movement behavior could explain their susceptibility to nematodes.

If infective juvenile foraging strategy was a significant factor in causing *D. coriaria* mortality then there would be higher susceptibility to an ambusher than a cruiser because *D. coriaria* is highly mobile in both the larval and adult stages. Of the nematodes assayed, *H. bacteriophora* is a cruiser and is more effective at finding sedentary hosts [22]; *S. carpocapsae* is an ambusher and is more effective at finding a mobile hosts [22]. *Steinernema feltiae* and *S. riobrave* both exhibit an intermediate behavior in the search continuum [23,40]. The observed mortality pattern is not consistent with nematode foraging behavior. Mortality caused by the ambusher *S. carpocapsae* was not significantly greater than the mortality from the cruiser *H. bacteriophora* (Figure 2). Mortality from *S. feltiae* and *S. riobrave*, the intermediates, was lower than cruiser and higher than the ambusher, respectively, not in between them. Thus, the results do not support the hypothesis that nematode foraging behavior explains *D. coriaria* susceptibility. This pattern holds for both the mortality data and frequency of nematode establishment.

The lack of dose response in *D. coriaria* mortality for any of the four nematode species may have been due to the simple arena and relatively high numbers of nematodes at even the lowest dose (Figure 1). For both *S. riobrave* and *S. carpocapsae* dose did not correlate with increased colonization of the host, however, colonization by both *H. bacteriophora* and *S. feltiae* was positively correlated with dose (Figure 2). Nematode foraging strategy is one hypothesis to explain this difference if *S. feltiae* searched more as a “cruiser” and *S. riobrave* searched more as an “ambusher.” Alternatively, differences in colonization patterns could be due to intrinsic host suitability for the four nematode species.

If *D. coriaria* mortality could be explained by the size of the infective juvenile, then *D. coriaria* would likely be more susceptible to the narrowest nematodes. The observed mortality does not correlate to infective juvenile size. The infective juvenile with the greatest width is *S. riobrave* at 28 microns [41]. With a mean body width of 26 microns, *S. feltiae* is the second largest infective juvenile [42]. Followed by *S. carpocapsae* at 25 microns and *H. bacteriophora* at 23 microns [42]. *Dalotia coriaria* showed higher susceptibility to *S. riobrave* and *S. carpocapsae* even though *H. bacteriophora* is the narrowest nematode. Thus, the size of natural openings did not prevent or allow certain nematode species entry into the host. The size of the beetle spiracles, anal, and oral openings is unknown.

*Dalotia coriaria* may be a poor host due to its relative size to nematodes. Nematodes need a host that is large enough to provide sufficient resources for reproduction. *Dalotia coriaria* is only 3–4 mm long and provides much less resources than *G. mellonella*, 12–20 mm in length. Also, from the perspective of the beetle, the infective juveniles are likely large enough—14% to 28% the size of the beetle—to be perceived and *D. coriaria* may have developed behaviors to avoid or groom nematodes before they can enter.

## 5. Conclusions

In conclusion, *D. coriaria* appears to be most likely compatible with applications of *S. feltiae* and *H. bacteriophora* and less compatible with *S. carpocapsae* and *S. riobrave*. The former two nematode species did not cause significantly higher morality than the control, and established in <20% of the beetles at or below the recommended rate for greenhouse applications. Thus, the results strongly suggest that biological control organisms *S. feltiae* and/or *H. bacteriophora* and *D. coriaria* could be applied at the same time to manage greenhouse pests such as fungus gnats and western flower thrips. This laboratory experiment presented a worse case scenario for the potential host (*D. coriaria*); a homogeneous habitat with limited potential refuges from a high population of foraging infective juveniles. The two-dimensional piece of vertical filter paper provided a simple environment for the infective juveniles to search and may have maximized infection. Since cruising nematodes can find hosts more effectively in a three-dimensional space than a two-dimensional space [23], potential negative interactions between *D. coriaria* and *S. carpocapsae* and *S. riobrave* should be confirmed in experiments that provide or approximate field conditions.
